# Application of an *E. coli* signal sequence as a versatile inclusion body tag

**DOI:** 10.1186/s12934-017-0662-4

**Published:** 2017-03-21

**Authors:** Wouter S. P. Jong, David Vikström, Diane Houben, H. Bart van den Berg van Saparoea, Jan-Willem de Gier, Joen Luirink

**Affiliations:** 1 Abera Bioscience AB, 11145 Stockholm, Sweden; 2 Department of Molecular Cell Biology, Section Molecular Microbiology, Faculty of Earth and Life Sciences, VU University, De Boelelaan 1085, 1081 HV Amsterdam, The Netherlands; 3 Xbrane Biopharma AB, 11145 Stockholm, Sweden; 4 Center for Biomembrane Research, Department of Biochemistry and Biophysics, Stockholm University, 10691 Stockholm, Sweden

**Keywords:** Inclusion bodies, Fusion tag, Insolubility, Aggregation, Heterologous protein production, *E. coli*, Signal peptide, Twin-arginine translocation pathway

## Abstract

**Background:**

Heterologous protein production in *Escherichia coli* often suffers from bottlenecks such as proteolytic degradation, complex purification procedures and toxicity towards the expression host. Production of proteins in an insoluble form in inclusion bodies (IBs) can alleviate these problems. Unfortunately, the propensity of heterologous proteins to form IBs is variable and difficult to predict. Hence, fusing the target protein to an aggregation prone polypeptide or IB-tag is a useful strategy to produce difficult-to-express proteins in an insoluble form.

**Results:**

When screening for signal sequences that mediate optimal targeting of heterologous proteins to the periplasmic space of *E. coli*, we observed that fusion to the 39 amino acid signal sequence of *E. coli* TorA (ssTorA) did not promote targeting but rather directed high-level expression of the human proteins hEGF, Pla2 and IL-3 in IBs. Further analysis revealed that ssTorA even mediated IB formation of the highly soluble endogenous *E. coli* proteins TrxA and MBP. The ssTorA also induced aggregation when fused to the C-terminus of target proteins and appeared functional as IB-tag in *E. coli* K-12 as well as B strains. An additive effect on IB-formation was observed upon fusion of multiple ssTorA sequences in tandem, provoking almost complete aggregation of TrxA and MBP. The ssTorA-moiety was successfully used to produce the intrinsically unstable hEGF and the toxic fusion partner SymE, demonstrating its applicability as an IB-tag for difficult-to-express and toxic proteins.

**Conclusions:**

We present proof-of-concept for the use of ssTorA as a small, versatile tag for robust *E. coli*-based expression of heterologous proteins in IBs.

**Electronic supplementary material:**

The online version of this article (doi:10.1186/s12934-017-0662-4) contains supplementary material, which is available to authorized users.

## Background

The Gram-negative bacterium *Escherichia coli* is by far the most popular host for the production of recombinant proteins in biotechnology because of the high expression levels that can be achieved, its rapid growth rate, its suitability for continuous and high-cell density culturing methods and general cost-effectiveness [1].

However, many recombinant polypeptides are prone to misfolding upon expression in bacteria due to the high rate of translation and the lack of cognate chaperones. Also, formation of disulphide bonds is not supported in the reducing environment of the bacterial cytosol, which may further compromise protein folding and stability. Many proteins are toxic to the host cell when expressed at high levels and inhibit cell growth or even induce cell death, leading to impaired protein production levels. Furthermore, at all stages during expression and purification, bacterial proteases may affect the yield of the desired product. Even when expression of properly folded soluble protein is achieved, purification and isolation of the recombinant proteins from the complex cytoplasm is difficult and labor intensive [2].

To address some of these problems, recombinant proteins may be routed to the periplasm, which provides an oxidative environment that is favorable for protein folding, disulphide bond formation and stability [3, 4]. To direct recombinant proteins to this compartment, they must be fused to an N-terminal signal sequence that mediates their targeting to and translocation across the bacterial inner membrane via either the Sec-system or the twin-arginine translocation (Tat) translocon, depending on the signal sequence chosen. Signal sequences are generally short (20–30 amino acid residues) and comprise three domains: a basic domain at the N-terminus, a central hydrophobic core, and a C-terminal domain that contains a cleavage site for Signal peptidase [5].

In many cases, overexpression of recombinant proteins in the cytosol and sometimes even in the periplasm leads to the formation of aggregates that consist almost exclusively of the recombinant protein [6]. Using light-microscopy, these aggregates or inclusion bodies (IBs) can be observed as large refractive bodies that are predominantly located at one or both cell poles [7, 8]. For long, IBs were considered to consist solely of unfolded or highly misfolded polypeptides. However, it now seems clear that, at least in specific cases, a significant part of IBs consists of properly folded and biologically active protein [9, 10]. Furthermore, expression in IBs seems an effective strategy to avoid some of the problems associated with expression of recombinant proteins in a soluble form. Proteins in IBs are largely resistant against degradation by host cell proteases and less likely to exert toxic effects. Moreover, due to their high density, IBs are easy to isolate from cell lysates by differential centrifugation, providing fast, robust and hence cost-efficient [11] protocols to obtain large amounts of relatively pure protein [12–14]. Improved methods for refolding partially denatured or incompletely folded recombinant proteins from IBs further contribute to the current interest in the deposition of recombinant protein in IBs [15, 16]. Rather than being seen as unwanted byproducts of protein production, IBs are nowadays regarded as functional nanoparticles with potential applications in for example biocatalysis, diagnostics, tissue engineering and drug delivery [17].

Some recombinant proteins form IBs already at relatively low expression levels while others remain completely soluble even at extremely high intracellular concentrations. Unfortunately, the propensity to form IBs is difficult to predict from the recombinant protein sequence. However, it has been shown that even intrinsically soluble proteins often accumulate in IBs when they are expressed as a fusion to an aggregation prone polypeptide [18]. These so-called IB-tags are convenient tools to produce difficult-to-express proteins.

Upon examination of signal sequences that mediate optimal targeting of recombinant proteins to the *E. coli* periplasm, we serendipitously found that the 39-amino acid long signal sequence of *E. coli* TMAO-reductase (ssTorA) promoted high-level expression of heterologous proteins in IBs, instead of facilitating translocation of these proteins across the cytoplasmic membrane. We present data that demonstrate the potential of ssTorA as a small, broadly applicable IB-formation tag for robust expression of recombinant proteins in IBs.

## Results

### Signal sequence-mediated periplasmic expression of heterologous proteins

**Fig. 1:**
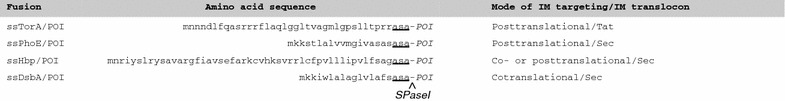
Schematic representation of *E. coli* signal sequence fusion constructs. Amino acid sequences of the signal sequences of *E. coli* TorA (ssTorA), PhoE (ssPhoE), Hbp (ssHbp) and DsbA (ssDsbA) as fused to a recombinant protein of interest (POI). A universal Ala-Ser-Ala sequence (*underlined*) for recognition by Signal peptidase I (SPaseI) replaces the three most C-terminal residues of each signal sequence

Periplasmic expression has shown to be an effective strategy to produce eukaryotic proteins in *E. coli*. Initially, we aimed to identify the most effective strategy to mediate the periplasmic localization of the recombinant human epidermal growth factor (hEGF). This small, 53 amino acid mitogen has various (potential) applications in wound healing and is difficult to produce at industrial scale in *E. coli* due to the presence of three disulphide bonds in the native protein [19]. To direct hEGF to the periplasm, fusion to various signal sequences for routing via different *E. coli* inner membrane targeting and translocation pathways was explored (Fig. 1). The fusion constructs were cloned under *tetA* promoter control [20] and expressed in *E. coli* K-12 strain MC4100 upon addition of the inducing agent anhydrotetracycline. After two hours, total cell samples of induced and non-induced cultures were analyzed by SDS-PAGE and Coomassie staining.

**Fig. 2:**
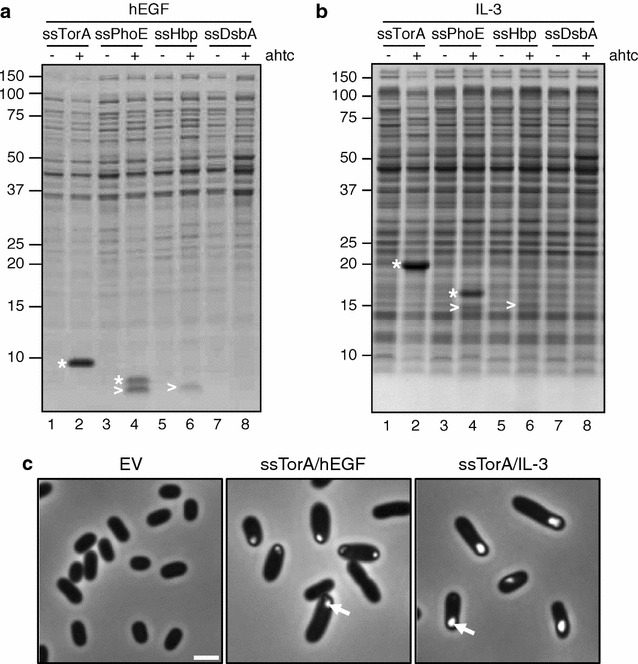
Expression of hEGF and IL-3 in IBs upon fusion to ssTorA. Expression of either hEGF (**a**) or IL-3 (**b**) upon fusion to the signal sequences (ss) of *E. coli* TorA, PhoE, Hbp or DsbA as indicated. *E. coli* MC4100 cells were grown to early log-phase when fusion protein expression was induced by addition of anhydrotetracycline (ahtc). Samples were withdrawn from the cultures at the time point of induction (−ahtc) and 2 h after induction (+ahtc). Cells were analyzed by SDS-PAGE and Coomassie staining. Non-processed (*) and processed (>) forms of the respective fusion proteins are indicated. Molecular mass (kDa) markers are indicated at the *left side* of the panels. **c** *E. coli* MC4100 cells carrying pIBA-ssTorA/hEGF, pIBA-ssTorA/IL-3 or an empty pASK-IBA3 vector (EV) grown and induced as described under **a** and **b** were analyzed by phase-contrast microscopy. Examples of inclusion bodies are indicated with an *arrow*. *Scale bar* 2 μm

Fusion of hEGF to the signal sequences of PhoE (ssPhoE) and Hbp (ssHbp), both mediating inner membrane translocation via the Sec-translocon [21, 22], resulted in the accumulation of a 6 kDa product in the cells (>) (Fig. 2a, lanes 4 and 6), which could be identified as hEGF by Western blotting (data not shown). The apparent molecular weight of the product suggested successful transport of the hEGF-chimeras to the periplasm and subsequent processing of the respective signal sequences by Signal peptidase I [23]. No clear hEGF product was observed upon fusion to the signal sequence of DsbA (ssDsbA), a cotranslationally targeted substrate of the Sec-translocon [24] (Fig. 2a, lane 8). This suggests that fusion to ssDsbA is ineffective in directing efficient periplasmic expression of hEGF and the ssDsbA/hEGF fusion protein is degraded. Fusion of hEGF to the signal sequence of the Tat-substrate TorA (ssTorA) [25, 26] yielded a ~10 kDa product corresponding to the non-processed form of ssTorA/hEGF. The product was expressed at remarkably high levels, being by far the most abundant protein in the cell lysate (Fig. 2a, lane 2). The lack of processing by Signal peptidase I suggests that hEGF failed to translocate to the periplasmic space of *E. coli* upon fusion to ssTorA. Similar results were obtained when investigating the signal sequence-mediated periplasmic expression of two other human proteins: interleukin-3 (IL-3) (Fig. 2b) and phospholipase A2 (Type V) (Pla2) (data not shown). Whereas chimeras comprising Sec-signal sequences showed (low-level) expression of processed IL-3 and Pla2, fusion to ssTorA produced high amounts of non-processed material in *E. coli* cells (Fig. 2b, lane 2; data not shown).

In conclusion, under the expression conditions used, fusion to ssTorA does not support translocation of recombinant proteins into the periplasm but rather induces high-level expression of the unprocessed form, presumably in the cytoplasm.

### The TorA signal sequence directs expression of heterologous proteins in inclusion bodies

To analyze the distribution of the accumulating ssTorA fusion proteins in more detail, *E. coli* cells were lysed and fractionated upon expression of ssTorA/hEGF (Additional file 1: Figure S1). A small fraction of the fusion protein accumulated in the membrane fraction and the soluble cytoplasmic fraction (Additional file 1: Figure S1, lanes 2 and 4). However, the vast majority of ssTorA/hEGF co-sedimented with the cellular debris upon low-speed centrifugation of the spheroplast lysate (Additional file 1: Figure S1, lane 5), suggesting that the protein was expressed in an insoluble form. As a control, the endogenous soluble cytoplasmic protein Trigger factor, the periplasmic protein DsbA and the (outer) membrane protein OmpA, were predominantly localized in the expected subcellular fraction, confirming the reliability of the fractionation procedure.

To examine the nature of TorA/hEGF aggregates, the low-speed pellet of a total lysate of *E. coli* cells expressing the protein was extracted with the detergent Triton X-100, which solubilizes membrane-associated material rather than aggregated proteins (Additional file 2: Figure S2). The majority of ssTorA/hEGF was detected in the pellet fraction (Additional file 2: Figure S2, lane 2) in contrast to the endogenous outer membrane protein OmpA, which was only detected in the supernatant fraction (Additional file 2: Figure S2, lane 3). Together, the data suggest that ssTorA/hEGF is expressed in dense cytoplasmic aggregates, also known as inclusion bodies. Consistently, phase-contrast microscopy of *E. coli* cells expressing ssTorA/hEGF showed clear polar foci (Fig. 2c). Similar structures were observed upon expression of ssTorA/IL-3 (Fig. 2c) and ssTorA/Pla2 (data not shown), indicating a more general propensity for heterologous proteins to accumulate in IBs upon fusion to ssTorA. Hence, ssTorA may be applicable as a fusion tag that enables efficient production of heterologous proteins in *E. coli* by directing their deposition in IBs.

### The TorA signal sequence directs expression of well-soluble *E. coli* proteins in inclusion bodies

**Fig. 3:**
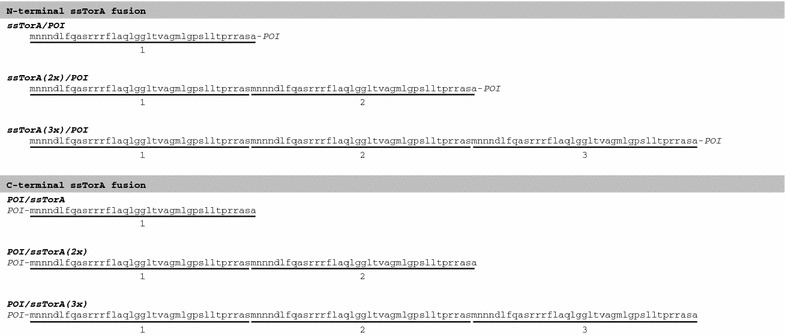
Schematic representation of ssTorA fusion constructs. Amino acid sequences of ssTorA when fused to the N-terminus or C-terminus of a recombinant protein of interest (POI), either in single, double, or triple format. As a result of the cloning procedure a threonine residue at the penultimate position of wild-type ssTorA is replaced by a serine. Furthermore, upon fusion of multiple ssTorA sequences, upstream ssTorA sequences lack the ultimate alanine residue

**Fig. 4:**
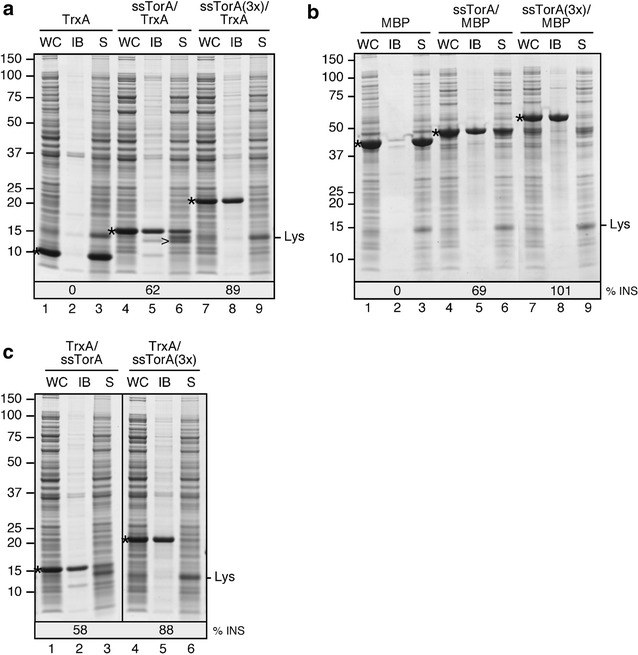
IB formation of TrxA and MBP upon fusion to ssTorA. **a** *E. coli* TOP10F’ cells expressing the indicated TrxA constructs were lysed and subjected to differential centrifugation to separate the inclusion bodies (IB)-containing insoluble fraction from the soluble cell fraction (S). Samples of both fractions and corresponding whole cell (WC) samples were analyzed by SDS-PAGE and Coomassie staining. All samples were derived from the same amount of cell material. **b** Cells expressing indicated MBP constructs processed and analyzed as described under **a**. **c** Cells expressing indicated TrxA constructs carrying ssTorA sequence(s) at the C-terminus processed and analyzed as described under **a**. Full-length expression products (*), a processed product of ssTorA/TrxA (>) and lysozyme added during the fractionation procedure (Lys) are indicated. Molecular mass (kDa) markers are indicated at the *left side* of the panels. At the* bottom* of the panels the relative amount of overexpressed protein in the insoluble fraction compared to the whole cell lysates is displayed (% INS) as determined by densitometric quantification of the respective Coomassie stained bands. The following calculation was used: (density of protein band in lane IB/density of protein band in lane WC)*100%

hEGF, IL-3 and Pla2 are eukaryotic proteins and therefore may have some intrinsic tendency to aggregate upon expression in the *E. coli* cytosol. In other words, they may constitute relatively easy targets for ssTorA-mediated production in IBs. To test the ability of ssTorA to promote aggregation of more challenging, intrinsically soluble proteins, ssTorA was fused to the N-terminus of the endogenous *E. coli* proteins Thioredoxin-1 (TrxA) and Maltose binding protein (MBP; lacking its signal sequence) using the same strategy as described for the fusion partners of eukaryotic origin (Fig. 3). Both TrxA and MBP remain soluble even at high intracellular concentrations of 35–40% of the total cellular protein content. In fact, they have been exploited as solubility-enhancing fusion partners of proteins that are otherwise insoluble upon expression in *E. coli* [27, 28]. IB formation of the resulting fusion proteins and corresponding ssTorA-less controls was analyzed using an IB sedimentation assay. Briefly, *E. coli* TOP10F’ cells expressing the proteins were broken by lysozyme treatment and sonication, after which the lysate was subjected to low-speed centrifugation to separate the dense cell material including IBs from the soluble proteins. The resulting fractions and a corresponding whole-cell lysate sample were analyzed by SDS-PAGE and Coomassie staining (Fig. 4a, b).

ssTorA-tagged versions of TrxA and MBP were expressed at levels similar to their non-tagged counterparts (Fig. 4a, b, cf. lanes 1 and 4). Judged by the intensity of the Coomassie stained bands, the proteins are by far the most abundant proteins in the expressing cells. Despite the high level of expression, the non-tagged proteins were completely recovered in the supernatant fraction (Fig. 4a, b, lane 3), confirming their solubility. In contrast, most ssTorA/TrxA (62%) and ssTorA/MBP (69%) was detected in the low-speed pellet (Fig. 4a, b, lane 5), indicating that the tagged proteins were largely insoluble upon overexpression. Notably, part of the population of ssTorA/TrxA recovered in the soluble fraction appeared unstable and was processed to a lower molecular weight form (>) (Fig. 4a, lane 6; Additional file 3: Figure S3), possibly due to a to non-cytosolic protease gaining access to the fusion protein upon disruption of the whole cells during the fractionation procedure.

**Fig. 5:**
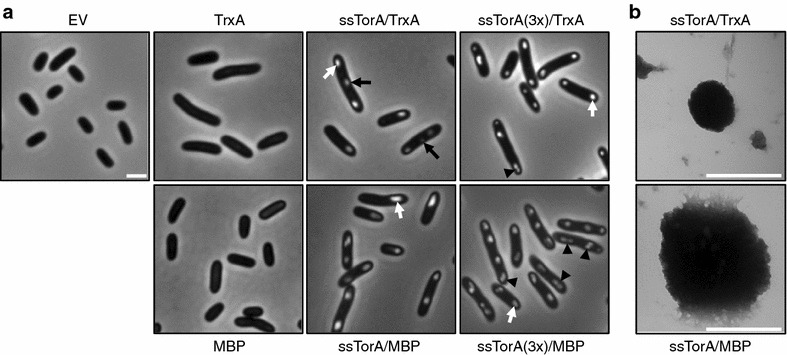
Microscopy analysis of IB formation. **a** *E. coli* TOP10F’ cells carrying an empty vector (EV) and cells expressing indicated proteins were analyzed by phase-contrast microscopy. Examples of IBs are indicated with a *white arrow*; examples of IBs seemingly formed at the new-pole of interconnected cells are indicated with a *black arrow*; examples of clustered IBs are indicated with a *black arrowhead*. *Scale bar* 2 μm. **b** Electron microscopy analysis of IBs isolated from TOP10F’ cells overexpressing indicated proteins. Representative IBs of average size are shown. *Scale bar* 500 nm

In agreement with the biochemical analysis, phase-contrast microscopy showed very prominent, mostly polar localized IBs in cells expressing ssTorA/TrxA and ssTorA/MBP, whereas no such structures were observed in cells expressing their non-tagged counterparts or carrying an empty vector (Fig. 5a). In agreement with observations by others [7], overexpression of IB-forming proteins seemed to interfere with cell division, resulting in elongated cells with IBs localized at both cell poles. Also, interconnected cells were present with IBs positioned at the old poles and emerging IBs at the interconnected new poles (Fig. 5a; *black arrow*). To get more insight into the nature and stability of the IBs, they were isolated from disrupted *E. coli* cells and subjected to an extensive washing regime with Triton X-100 detergent to remove excess membrane material, urea to break low affinity protein interactions and high-salt to break potential electrostatic protein interactions. Subsequent analysis by electron microscopy revealed a homogenous population of particulate structures for both fusion proteins (Additional file 4: Figure S4). With estimated average diameters of 750 and 350 nm respectively (Fig. 5b; Additional file 4: Figure S4), ssTorA/MBP IBs appeared significantly larger than the ssTorA/TrxA IBs. Remarkably, whereas ssTorA/TrxA IBs overall had a characteristic spherical appearance, many IBs isolated from by ssTorA/MBP expressing cells displayed flat sides (Additional file 4: Figure S4). Possibly, parts of these IBs broke off due to the extensive washing regime applied during isolation, which involved several rounds of sonication. In any case, this analysis confirmed that genuine, stable IBs were generated upon fusion to ssTorA, although the nature or size of the fused cargo protein can have a considerable influence on the size and appearance of the IBs formed. The finding that fusion of ssTorA to the N-terminus of otherwise highly soluble endogenous *E. coli* proteins in *E. coli* induces their deposition in IBs underscores the potential of ssTorA as a universal IB fusion tag.

### Enhanced efficiency of IB-formation upon fusion to multiple ssTorA sequences

Despite the small size of the tag, remarkably efficient IB-formation of target proteins was observed upon attachment of a single ssTorA sequence. In an attempt to further improve the IB-formation process, the N-termini of TrxA and MBP were equipped with triple tandem repeats of ssTorA (see Fig. 3). The efficiency of insoluble expression was analyzed using the sedimentation assay described above. In contrast to the corresponding single-ssTorA versions, the triple-ssTorA carrying chimeras ssTorA(3x)/TrxA (Fig. 4a, lane 8), and ssTorA(3x)/MBP (Fig. 4b, lane 8), were exclusively detected in the IB-containing low-speed pellet. Notably, although the steady-state expression levels of the triple-ssTorA chimeras appeared slightly reduced compared to their cognate single-ssTorA variants, the absolute amounts of non-soluble material produced appeared significantly increased for these constructs (Fig. 4a, b, cf. lanes 5 and 8). Fusion of double ssTorA tandem repeats (see Fig. 3) produced very similar results (Additional file 5: Figure S5A, B), reducing the length of the tag needed to reach optimal IB-formation.

Phase-contrast microscopy was performed to analyze the effect of multiple tag attachment on the appearance of IBs (Fig. 5a). Expression of triple-tagged fusion proteins seemed to yield more IBs per cell compared to proteins carrying one tag. In contrast to cargos carrying one tag, many examples were found of cells that contained structures that seemed to represent clusters of multiple of IBs (*black arrow heads*) rather than singular IBs. The origin of this remarkable observation is as yet unclear and subject to further analysis. Nevertheless, the collective data show that the amounts of insoluble protein produced per cell can be improved by fusing multiple ssTorA tags to target proteins, rather than one tag.

### Successful IB-formation upon fusion of ssTorA to the C-terminus of TrxA

For some target proteins or applications fusion of the tag to the C-terminus might be more appropriate, for example when the biological activity of proteins produced in IBs or refolded from IBs is dependent on a non-modified N-terminus. To investigate whether a C-terminal location of the tag is compatible with IB-formation, a single-or triple-ssTorA sequence was fused downstream of TrxA (see Fig. 3). The resulting TrxA/ssTorA chimeras were expressed at a level comparable to TrxA chimeras carrying N-terminal ssTorA (cf. Fig. 4a, lane 4, c, lane 1). Furthermore, very similar to the N-terminal ssTorA (Fig. 4a, lane 5), the C-terminal fusion appeared to render TrxA mostly insoluble using an IB sedimentation assay (Fig. 4c, lane 2). Upon fusion of two (Additional file 5: Figure S5C) or three tags almost all material was detected in the pellet (Fig. 4c, lane 5), demonstrating enhanced insolubility under these conditions, again similar to the corresponding N-terminal ssTorA fusions (Fig. 4a, lane 8). In conclusion, ssTorA functions as an IB-tag at the N-terminus and the C-terminus of a target protein with approximately equal efficiency.

### ssTorA drives IB formation in *E. coli* K-12 and B strains

*Escherichia coli* K-12 and B strains are both employed in industrial protein production processes. To exclude that ssTorA-mediated IB-formation of target proteins is specific for expression in *E. coli* K-12 strains, like the strains MC4100 and TOP10F’ used thus far, we analyzed expression and IB-formation of MBP and ssTorA/MBP under *tetA* promoter control in *E. coli* B strain BL21(DE3) (Additional file 6: Figure S6). In this background, the expression levels of MBP and ssTorA/MBP were comparable to TOP10F’. The efficiency of insoluble expression of ssTorA/MBP in BL21(DE3) appeared even slightly higher than in TOP10F’ indicating that the ssTorA IB-tag is functional in various *E. coli* strains.

### ssTorA-mediated production of unstable and toxic proteins

**Fig. 6:**
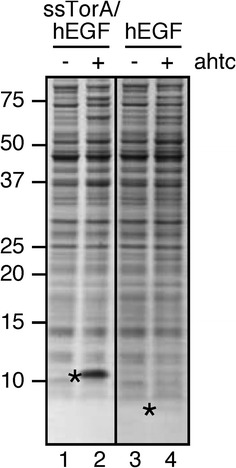
Expression of hEGF in the absence and presence of fused ssTorA. *E. coli* HDB37 cells were grown to early log-phase when expression of either hEGF or ssTorA/hEGF was induced by addition of anhydrotetracycline (ahtc). Samples were withdrawn from the cultures at the time point of induction (−ahtc) and 2 h after induction (+ahtc). Cells were analyzed by SDS-PAGE and Coomassie staining. Expression products hEGF and ssTorA/hEGF are indicated (*). Molecular mass (kDa) markers are indicated at the *left side* of the panels

ssTorA may be exploited for the production of proteins that are difficult to produce when expressed in *E. coli* in a soluble form. To investigate whether ssTorA can enhance the expression of proteins that are unstable in *E. coli*, it was fused to the N-terminus of hEGF, which is poorly expressed in *E. coli* and hardly detectable in a total cell lysate (Fig. 6, lane 4). As observed before (Fig. 2a, lane 2), ssTorA/hEGF expressed under identical conditions appeared the most abundant protein in the *E. coli* lysate (Fig. 6, lane 2). Most likely, insoluble expression of hEGF upon fusion of ssTorA renders the protein (largely) inaccessible to proteolytic degradation in the *E. coli* cytoplasm resulting in dramatically enhanced expression levels.

**Fig. 7:**
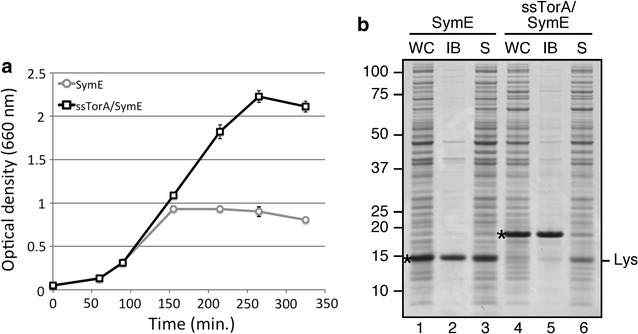
Expression and inclusion body formation of the toxin SymE in the absence and presence of fused ssTorA. **a** Growth curves for *E. coli* TOP10F’ cells expressing SymE or ssTorA/SymE as indicated. Cells were grown to an OD_660_ of 0.3 when expression of SymE or ssTorA/SymE was induced by addition of anhydrotetracycline. *Error bars* indicate standard deviation; n = 3. **b** Inclusion body formation of SymE and ssTorA/SymE in the cultures described under **a** analyzed and displayed as described in the legend to Fig. 4

An alternative cause of impaired recombinant protein expression is toxicity of the native protein through interference with the physiology of the expression host, leading to growth defects or even cell death. To analyze the expression of toxic proteins through the ssTorA-fusion approach, ssTorA was fused to the cytoplasmic *E. coli* toxin SymE that is part of the SymE-SymR toxin-antitoxin system [29]. Overexpression of this protein has been described to decrease protein synthesis, provoke RNA degradation and consequently inhibit growth [30]. Indeed, growth of *E. coli* TOP10F’ cells was strongly inhibited upon expression of SymE under control of a *tetA* promoter with the culture reaching an optical density (OD_660_) of only 0.9 approximately 3 h after induction of SymE expression (Fig. 7a). In contrast, upon tagging of SymE with the ssTorA, growth was restored, with the culture reaching an OD_660_ of more than 2.0. To investigate how the influence on cell growth relates to the protein expression and solubility, cell samples of SymE and ssTorA/SymE were taken 2 h after induction and subjected to the IB sedimentation assay (Fig. 7b). The majority of non-tagged SymE appeared present in the supernatant fraction (Fig. 7b, lane 3) whereas ssTorA/SymE was almost exclusively present in the pellet containing insoluble material (Fig. 7b, lane 4). Apparently, aggregation of ssTorA/SymE alleviates the toxic effects associated with overexpression of SymE in soluble form. Taken together, the data suggest that fusion to ssTorA is an effective strategy to produce difficult-to-express proteins, such as unstable and toxic proteins, in *E. coli*.

## Discussion

Here, we present a proof-of-concept study that demonstrates the potential of ssTorA as a tool for high-level insoluble protein expression, to allow for straightforward and cost-efficient recombinant protein production. We show that fusion of ssTorA mediates aggregation of all tested proteins including those that are soluble even when expressed at high intracellular concentrations (TrxA and MBP). Conveniently, ssTorA is functional at both the N-terminus and C-terminus of target proteins and can be employed in *E. coli* K-12 as well as B strains. An even stronger aggregation propensity was observed upon fusion of multiple ssTorA sequences in tandem allowing almost complete aggregation even of the highly soluble TrxA and MBP proteins. To demonstrate its applicability, ssTorA was successfully used to overproduce intrinsically unstable hEGF in *E. coli* and to improve growth of host bacteria upon expression of the toxic SymE protein.

The use of fusion tags to facilitate the insoluble expression of target proteins has been reported previously. Examples of insolubility tags include *trp*ΔLE [31], ketosteroid isomerase [32], β-galactosidase [33], PagP [34], EDDIE [35], ELK16 [36], GFIL8 [37], PaP3.30 [38], TAF12-HFD [39] and the F4 fragment of PurF [40]. Most described tags comprise relatively large sequences [31, 34, 35, 38, 40] that may adversely affect the yield of the target protein or compromise downstream applications. Smaller IB-tags have been presented but were used for the production of short polypeptides [39] or the efficiencies of IB formation and final yield of protein were difficult to evaluate [36, 41]. In addition, some of the target proteins analyzed in combination with small IB-tags seemed to possess an intrinsic tendency to form aggregates in the *E. coli* cytoplasm, making it difficult to evaluate the added value of the IB-tag [36, 37, 41, 42]. Here, we demonstrated that fusion of a single 39 amino acid ssTorA tag is sufficient to provoke efficient accumulation of the sizeable proteins TrxA and MBP into IBs, whereas no significant IB formation was observed for the corresponding non-tagged proteins expressed at similar levels (Fig. 4). Virtually complete deposition in IBs could be achieved upon attachment of a second (Additional file 5: Figure S5) or third tag (Fig. 4). Given that both TrxA and MBP are extensively used as fusion partner to enhance the solubility of heterologous proteins in *E. coli* [43], these results are striking and attest to the usefulness of ssTorA as an IB-tag.

The interest in IBs has been revived by recent studies showing that aggregated proteins can retain native-like conformations. Rather than being amorphous aggregates of disordered proteins, IBs show a structured β-sheet organization and bind amyloid specific dyes [44]. Furthermore, in some cases IBs were shown to contain a significant portion of properly folded and biologically active protein [17], showing potential for novel industrial and biomedical applications. Interestingly, fusion of a triple ssTorA tag to GFP yielded IBs that emitted a fluorescence signal, hence containing properly folded GFP (Additional file 7: Figure S7). This observation alludes to a compatibility of the ssTorA tag with the production of bioactive IBs. The relative amounts of functional protein present in these IBs and the question whether the production of bioactive IBs is a more general feature of the ssTorA tag are subjects of further research.

The observation that ssTorA promotes aggregation of fused proteins was very surprising and counterintuitive. Signal sequences normally mediate membrane targeting of fused proteins that are kept in a soluble and translocation competent conformation by interaction with cytosolic chaperones such as SecB and DnaK [45]. TorA belongs to a family of proteins that are equipped with a ‘twin-arginine’ signal sequence that contains a distinctive SRRxFLK amino acid motif. These signal sequences mediate protein transport via the Tat-pathway, which is specialized in the translocation of fully folded substrates across the cytoplasmic membrane [46]. An intrinsic quality control mechanism, sometimes involving specific signal sequence binding chaperones (e.g. TorD for ssTorA) [47], prevents targeting of proteins with a non-native conformation [48]. IL-3, Pla2, hEGF (all eukaryotic proteins) are likely to fold incorrectly in the *E. coli* cytoplasm and may be incompatible with Tat-export. However, while TrxA and MBP are compatible with Tat-mediated translocation when fused to ssTorA [49, 50], we observed that overexpression of ssTorA/TrxA and ssTorA/MBP resulted in IB formation. Surprisingly, the overexpression conditions used even caused efficient IB formation of full-length TorA (mature TorA coupled to its cognate ssTorA) (Additional file 8: Figure S8). Apparently, ssTorA-dependent IB formation is not per se driven by an incompatibility of cargo proteins with the Tat-dependent export pathway.

Fusion to ssTorA has been successfully used to transport heterologous proteins such as GFP across the *E. coli* inner membrane into the periplasm [51]. Instead, in the present work we demonstrate efficient IB-formation of cargos upon attachment of ssTorA, including GFP (see Additional file 7: Figure S7). The discrepancy between these results may find its origin in the different expression regimes used. Export of ssTorA/GFP to the periplasmic space seems to rely on conditions ensuring modest expression levels such as the use of reduced growth temperatures or sub-saturating concentrations of inducing agent [51, 52]. In contrast, we overexpressed proteins to high cellular abundance (e.g. see Figs. 2, 4) from a high-copy number vector, under control of the reportedly very strong and robust *tetA* promoter [53, 54]. Furthermore, we made use of saturating amounts of anhydrotetracycline to induce expression. Conceivably, these high-expression conditions are key to mediate efficient insoluble expression of heterologous proteins using ssTorA. It should be mentioned that successful IB formation is not restricted to *tetA* driven expression since high-level expression of full-length TorA from the *ara*BAD promoter (Additional file 8: Figure S8) and *lac*UV5-controlled expression of a fusion comprising ssTorA and the 143 kDa autotransporter Hemoglobin protease [55] (Additional file 9: Figure S9) also resulted in efficient IB-formation.

What is the molecular basis of ssTorA-mediated IB formation? IBs form when proteins exposing hydrophobic segments specifically cluster in a nucleation-dependent mechanism [56, 57]. Nucleation of aggregation may start when excess ssTorA-fusions, carrying improperly folded and translocation-incompatible cargos, arrive at the inner membrane and stall in an unproductive attempt to get translocated via the Tat-machinery. In an alternative scenario, overexpression of ssTorA-fusion proteins leads to an imbalance between ssTorA and its cognate chaperone TorD, which is poorly expressed in regular growth media lacking elevated levels of the compound TMAO [58]. This may lead to interaction between the relatively hydrophobic TorA signal sequences [59, 60] and support the nucleation required for IB formation. The additive effect of the attachment of multiple signal sequences is in line with this scenario. Alternatively, overexpression of ssTorA may cause titration of cytosolic chaperones like DnaK and GroEL with reported (Tat-)signal sequence- binding properties [61, 62]. Given the generic role of these chaperones in preventing protein misfolding [63], their sequestration by ssTorA could result in aggregation of fused cargo proteins. In a fourth scenario, fusion of ssTorA forces a conformation upon the fused cargo protein that is prone to aggregation. Current research in our laboratory is focused on resolving which of these scenarios is correct.

## Conclusions

We have identified the signal sequence of *E. coli* TorA as a small and robust, versatile fusion tag that mediates efficient IB formation of proteins upon overexpression in *E. coli*. As such, the ssTorA IB-tag seems a valuable tool for the time- and cost-efficient production of proteins in general and the production of difficult-to-express proteins in particular.

## Methods

### Strains, media and growth conditions

*Escherichia coli* K-12 strains TOP10F’ (Invitrogen, UK), MC4100 [64] and HDB37 (MC4100 *ara*Δ) [65], as well as *E. coli* B strain BL21(DE3) (Novagen, Germany) were used for protein expression, as indicated. Cells were grown in LB medium in a shake incubator in the presence of ampicillin (100 μg/ml). Cultures were grown at 37 °C in flasks in a shake incubator (200 rpm) using a 5:1 flask:culture volume ratio.

### Reagents and sera

The Rapid Dephos & Ligation Kit was obtained from Roche Applied Science. Phusion High Fidelity DNA polymerase was purchased from New England Biolabs (NEB). DNA restriction enzymes were from Roche or NEB. Lumi-Light Western Blotting Substrate was supplied by Roche. Skim milk was purchased from Thermo Fisher Scientific. Lysozyme and all other chemicals were purchased from Sigma-Aldrich. The rabbit polyclonal antisera against recombinant hEGF (ab9697) and TrxA (T0803) were from Abcam and Sigma Aldrich, respectively. Rabbit polyclonal antisera against trigger factor and OmpA were from our own lab collections, whereas the polyclonal antiserum against DsbA was from the sera collection of the laboratory of J. Beckwith (Harvard Medical School, Boston, MA, USA).

### Plasmid construction

The plasmids and primers used in this study can be found in Additional file 10: Table S1 and Additional file 11: Table S2, respectively. Details on the construction of plasmids can be found in Additional file 12 (Supplement methods).

### IB sedimentation assay

To separate IBs from the soluble cell content, a culture volume containing the number of cells that gives an optical density (660 nm) of 1.5 in a 1 ml suspension was subjected to centrifugation. The pelleted intact cells were resuspended in 750 μl ice-cold lysis buffer (5 mM Tris–HCl, pH 7.6, 1 mM EDTA, 100 mM NaCl). Lysozyme was added to a final concentration of 17 ng/ml and cells were incubated on ice for 15 min. Subsequently, the cells were disrupted by freeze-thawing and tip sonication (Branson Sonifier 250). The resulting lysate was centrifuged (4500×*g*, at 4 °C for 10 min) to sediment IBs and other dense, insoluble material. The resulting pellet was subjected to SDS-PAGE analysis directly, whereas the supernatant was trichloroacetic acid precipitated first. Intact cells directly subjected to SDS-PAGE analysis served as a control for total cell content.

### General protein expression and analysis

Plasmid-based protein expression was induced using anhydrotetracycline (0.2 µg/ml) (IBA GmbH) when cell cultures reached an OD_660_ of approximately 0.3. For analysis, cells and cell-fractions were resuspended in SDS-sample buffer (125 mM Tris–HCl pH 6.8, 4% SDS, 20% glycerol, 0.02% bromophenolblue, 83 mM DTT) and incubated at 96 °C for 10 min. Proteins were analyzed by SDS-PAGE and Coomassie Brilliant Blue G (Jansen Chimica) staining or Western blotting. Commercial Bis–Tris NuPAGE (Invitrogen) or TGX gels (Biorad) were used where appropriate. Imaging and densitometric quantification of Coomassie-stained gels was carried out using a GS-800 densitometer (Biorad) in combination with Quantity-One software (Biorad). For Western blotting, proteins were transferred to nitrocellulose membranes (GE Healthcare). Membranes were blocked using buffer TBS-T (TBS, 0.1% Tween-80) containing with 5% (w/v) skim milk. Membranes were incubated with primary antibodies in TBS-T containing 2.5% (w/v) skim milk for 1 h at room temperature. After washing with TBS-T, the membranes were incubated with peroxidase-conjugated anti-rabbit IgGs (1:10,000) (Rockland Immunochemicals) in TBS-T containing 2.5% (w/v) skim milk for 1 h at room temperature. After washing with TBS-T, immunoreactive bands were detected by chemiluminescence using Lumi-Light Western blotting substrate (Roche). Signals were captured using a Fluor-S MultiImaging system (Biorad).

### Phase-contrast microscopy

Unless stated otherwise, the following procedure was used to perform phase-contrast microscopy: Cells were first fixed by incubation in PBS containing 2.8% formaldehyde and 0.04% glutaraldehyde at room temperature for 15 min. The cells were kept cold during all subsequent procedures. To remove residual formaldehyde and glutaraldehyde, the cells were collected by low-speed centrifugation, transferred to a new tube and resuspended in fresh PBS. After three additional washings in PBS, the cells were resuspended in PBS before immobilization on 1% agarose in water slab-coated object glasses as described [66]. Samples were photographed with a CoolSnap *fx* (Photometrics) CCD camera mounted on an Olympus BX-60 fluorescence microscope through a UPLANFl 100 × /1.3 oil objective. Images were acquired with Micro-Manager (http://www.micro-manager.org/) with direct output of the desired hyperstack structure for ImageJ by Wayne Rasband (http://imagej.nih.gov/ij/).

### Isolation and electron microscopy of IBs

To isolate IBs for EM analysis, cells were collected by centrifugation 2 h and 30 min after induction of protein expression. The cells were resuspended in Lysis buffer (10 mM Tris–HCl pH8, 1 mM EDTA, 5 μg/ml lysozyme), incubated at 37 °C for 1 h and 15 min, and lysed using a tip sonicator (Branson Sonifier 250). The resulting lysate was subjected to centrifugation (15,000×*g*, 15 min) to sediment the IBs. To remove contaminants, the pelleted IB material was subjected to a number of consecutive washing steps: The IBs were resuspended in 10 mM Tris–HCl pH8, 1 mM EDTA using tip sonication, after which the suspension was mixed with an equal volume of Triton Wash Buffer (10 mM Tris–HCl pH8, 1 mM EDTA, 2% Triton X-100) and incubated at room temperature for 1 h. IBs were collected by centrifugation (15,000×*g*, 15 min) and resuspended in 10 mM Tris–HCl pH8 using tip sonication. After addition of an equal volume of Urea Wash buffer (10 mM Tris–HCl pH8, 2 M Urea) the suspension was incubated at room temperature for 1 h. IBs were again collected by centrifugation (15,000×*g*, 15 min), resuspended in 10 mM Tris–HCl pH8 using sonication, and an equal volume of High Salt Wash buffer (10 mM Tris–HCl pH8, 2 M NaCl) was added. IBs were then sedimented by centrifugation (15,000×*g*, 25 min), resuspended in 10 mM Tris–HCl pH8 by sonication, again sedimented by centrifugation (15,000×*g*, 25 min), and resuspended in PBS containing 15% glycerol.

For electron microscopy analysis, the IBs were spotted on carbon coated Formvar grids (FCF300-Ni, Aurion) for 5 min, and washed 5 times on water droplets. Negative staining was performed using 3.5% uranyl acetate for 5 min, after which excessive staining was removed and the grids were air dried. Samples were analysed at 120 kV on a Tecnai 12 (FEI) microscope.

## Additional files

**Additional file 1: Figure S1.** Subcellular fractionation of cells expressing ssTorA/hEGF.

**Additional file 2: Figure S2.** Differential Triton X-100 extraction of insoluble ssTorA/hEGF material.

**Additional file 3: Figure S3.** Occurrence of a proteolytic product of ssTorA/TrxA in the soluble fraction upon IB isolation.

**Additional file 4: Figure S4.** Electron microscopy analysis of IB formation.

**Additional file 5: Figure S5.** IB formation upon fusion to a dual ssTorA-tag.

**Additional file 6: Figure S6.** ssTorA-mediated inclusion body formation in *E. coli* K-12 and B strains.

**Additional file 7: Figure S7.** Expression of a ssTorA(3x)/GFP fusion protein yields fluorescent IBs.

**Additional file 8: Figure S8.** Inclusion body formation upon expression of full-length TorA.

**Additional file 9: Figure S9.** Inclusion body formation upon expression of Hbp.

**Additional file 10: Table S1.** Plasmids used in this study.

**Additional file 11: Table S2.** Primers used in this study.

**Additional file 12.** Supplement methods.

## Abbreviations

IB: inclusion body
IBs: inclusion bodies
ssTorA: signal sequence of TorA
Tat: twin-arginine translocation
SDS-PAGE: sodium dodecyl sulphide-polyacrylamide gel electrophoresis
