# Supplementary material for: Application of an E. coli signal sequence as a versatile inclusion body tag
Source: Microb Cell Fact. 2017 Mar 21;16:50. doi: 10.1186/s12934-017-0662-4 (PMC5359840; doi:10.1186/s12934-017-0662-4)
Supplement: Supplementary file 1 — Additional file 1: Figure S1. Subcellular fractionation of cells expressing ssTorA/hEGF. [file 12934_2017_662_MOESM1_ESM.pdf]

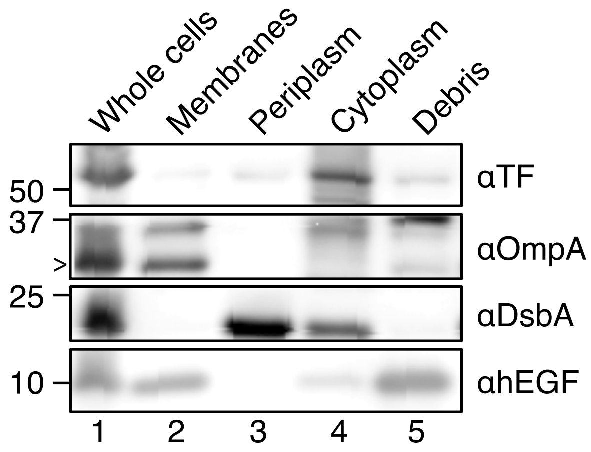

Fig. S1. Subcellular fractionation of cells expressing ssTorA/hEGF. Subcellular fractionation. *E. coli* TOP10F' cells expressing ssTorA/hEGF were grown in the presence of 5 mM $MgCl_2$ to an OD_{660} of 0.3, after which protein expression was induced with anhydrotetracycline for 30 min. Cells were collected by centrifugation (5,000 x g for 5 min) and kept cold during the remainder of the procedure. Cells were resuspended in ice-cold 10 mM Tris, pH 8, containing 20 % sucrose to an OD_{660} of 15. To induce spheroplast formation, 1/8 volume of lysozyme solution (2 mg/ml; 0.1 M EDTA) was added and spheroplasting was monitored by light microscopy. When more than 60% of the cells were converted into spheroplast the process was stopped by addition of $MgCl_2$ to a final concentration of 10 mM. The periplasmic content was separated from the spheroplasts by centrifugation at 6,000 x g for 2 min. To remove residual membrane material, the resulting supernatant fraction containing the periplasmic content was centrifuged once more at 115,000 x g for 10 min using a Beckmann TLA 120.2 rotor. The spheroplast pellet was resuspended in 100 μl spheroplast buffer (0.1 M Tris-HCl, pH 8.0, 250 mM sucrose). Subsequently, 900 μl of ice-cold distilled water was added and the speroplasts were lysed by tip sonication (Branson sonifier 250). The debris was removed from the resulting lysate by centrifugation at 4,000 x g for 10 min. To separate the cytoplasmic fraction from the membranes, the clarified spheroplast lysate was centrifuged at 355,000 x g for 30 min using a Beckmann TLA 120.2 rotor. Samples were analyzed by SDS-PAGE and Western blotting using the specified antisera. Whole cells and pellet fractions containing the cell debris or membranes were analyzed directly, whereas soluble supernatant fractions containing the periplasmic and cytoplasmic proteins were first subjected to trichloroacetic acid precipitation. Samples derived from equivalent amounts of cell material were analyzed. A folded, SDS-resistant form of OmpA with a reduced apparent molecular weight [1] is indicated (>) at the left side of the concerning panel. Molecular weight markers (kDa) are indicated at the left side of the panels.

Reference

1. Reithmeier RA, Bragg PD: **Purification and characterization of heat-modifiable protein from the outer membrane of *Escherichia coli*.** *FEBS Lett* 1974, **41:**195-198.
