# Supplementary material for: Application of an E. coli signal sequence as a versatile inclusion body tag
Source: Microb Cell Fact. 2017 Mar 21;16:50. doi: 10.1186/s12934-017-0662-4 (PMC5359840; doi:10.1186/s12934-017-0662-4)
Supplement: Supplementary file 2 — Additional file 2: Figure S2. Differential Triton X-100 extraction of insoluble ssTorA/hEGF material. [file 12934_2017_662_MOESM2_ESM.pdf]

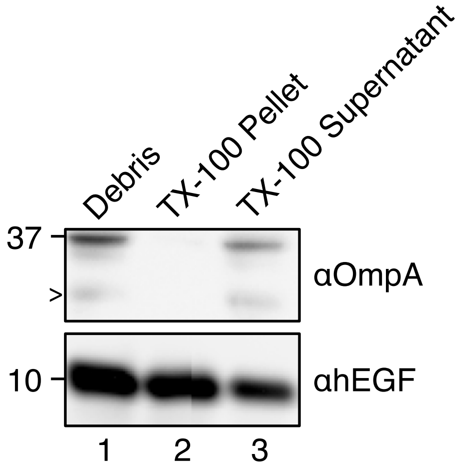

Fig. S2. Differential Triton X-100 extraction of insoluble ssTorA/hEGF material. *E. coli* TOP10F' cells carrying pIBA-ssTorA/hEGF were grown in the presence of 5 mM MgCl2 to an OD_{660} of 0.3 when protein expression was induced with anhydrotetracycline for 30 min. Collected cells were resuspended in 600 ul of lysis buffer (50 mM Tris-HCl, pH7.6, 100 mM NaCl, 1 mM EDTA) and disrupted by tip sonication (Branson sonifier 250). The cell debris was isolated by centrifugation at 4,500 x g for 10 min and resuspended in extraction buffer (50 mM Tris-HCl, pH7.6, 100 mM NaCl, 1 mM EDTA, 1% Triton X-100). Subsequently, the Triton-insoluble material was isolated by centrifugation at 4,500 x g for 10 min and washed once with lysis buffer. The protein content of the debris, the washed Triton-insoluble material (*pellet*) and the Triton X-100 extract (*supernatant*) were analyzed by SDS-PAGE and Western blotting using the indicated antisera. Samples derived from equivalent amounts of cell material were analyzed. A folded, SDS-resistant form of OmpA with a reduced apparent molecular weight [1] is indicated (>) at the left side of the concerning panel. Molecular weight markers (kDa) are indicated at the left side of the panels.

Reference

1. Reithmeier RA, Bragg PD: **Purification and characterization of heat-modifiable protein from the outer membrane of *Escherichia coli*.** *FEBS Lett* 1974, **41:**195-198.
