# Supplementary material for: Application of an E. coli signal sequence as a versatile inclusion body tag
Source: Microb Cell Fact. 2017 Mar 21;16:50. doi: 10.1186/s12934-017-0662-4 (PMC5359840; doi:10.1186/s12934-017-0662-4)
Supplement: Supplementary file 3 — Additional file 3: Figure S3. Occurrence of a proteolytic product of ssTorA/TrxA in the soluble fraction upon IB isolation. [file 12934_2017_662_MOESM3_ESM.pdf]

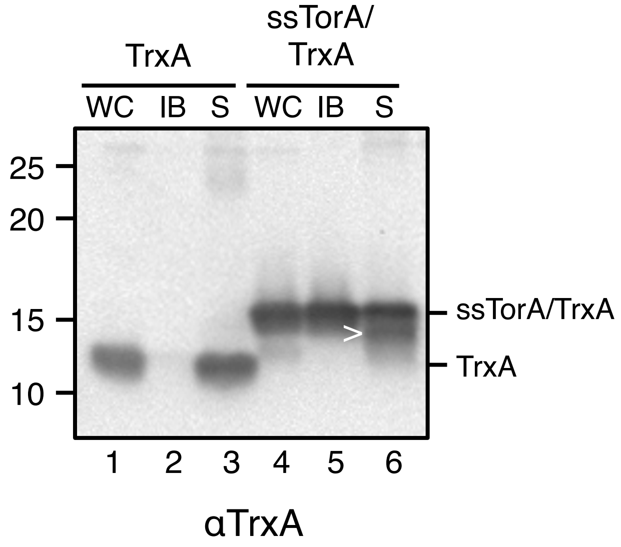

Fig. S3. Occurrence of a proteolytic product of ssTorA/TrxA in the soluble fraction upon IB isolation. Cells expressing TrxA or ssTorA/TrxA were subjected to a IB sedimentation assay as described in the legend to Fig. 4. Samples were analyzed by SDS-PAGE and Western blotting using a polyclonal antiserum against TrxA. Samples derived from equivalent amounts of cell material were analyzedA proteolytic product of ssTorA/TrxA that emerges specifically in the soluble fraction upon IB isolation (lane 6) is indicated (>). Molecular weight markers (kDa) are indicated at the left side of the panel.
