# Supplementary material for: Application of an E. coli signal sequence as a versatile inclusion body tag
Source: Microb Cell Fact. 2017 Mar 21;16:50. doi: 10.1186/s12934-017-0662-4 (PMC5359840; doi:10.1186/s12934-017-0662-4)
Supplement: Supplementary file 4 — Additional file 4: Figure S4. Electron microscopy analysis of IB formation. [file 12934_2017_662_MOESM4_ESM.pdf]

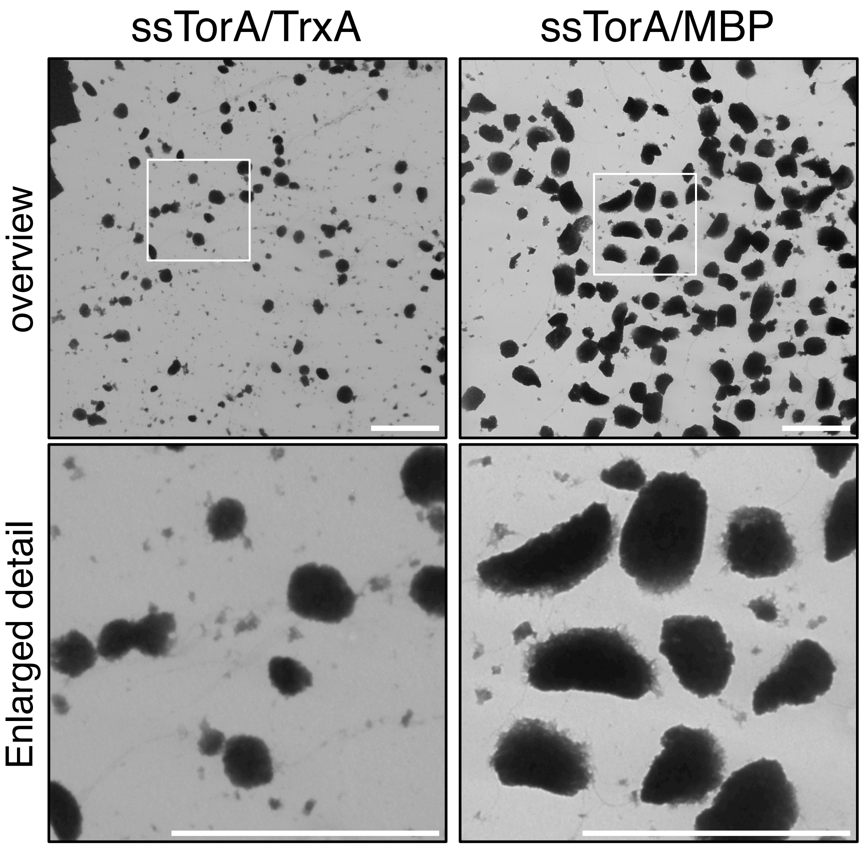

Fig. S4. Electron microscopy analysis of IB formation. Suspensions of IBs isolated from TOP10F' cells overexpressing ssTorA/TrxA, or ssTorA/MBP were analyzed by electron microscopy. Top panels display overview images of IB suspensions. Bottom panels display enlargements of boxed areas of corresponding top panels. White scale bars in the lower right-hand corner of the panels are 2 μm.
