# Supplementary material for: Application of an E. coli signal sequence as a versatile inclusion body tag
Source: Microb Cell Fact. 2017 Mar 21;16:50. doi: 10.1186/s12934-017-0662-4 (PMC5359840; doi:10.1186/s12934-017-0662-4)
Supplement: Supplementary file 5 — Additional file 5: Figure S5. IB formation upon fusion to a dual ssTorA-tag. [file 12934_2017_662_MOESM5_ESM.pdf]

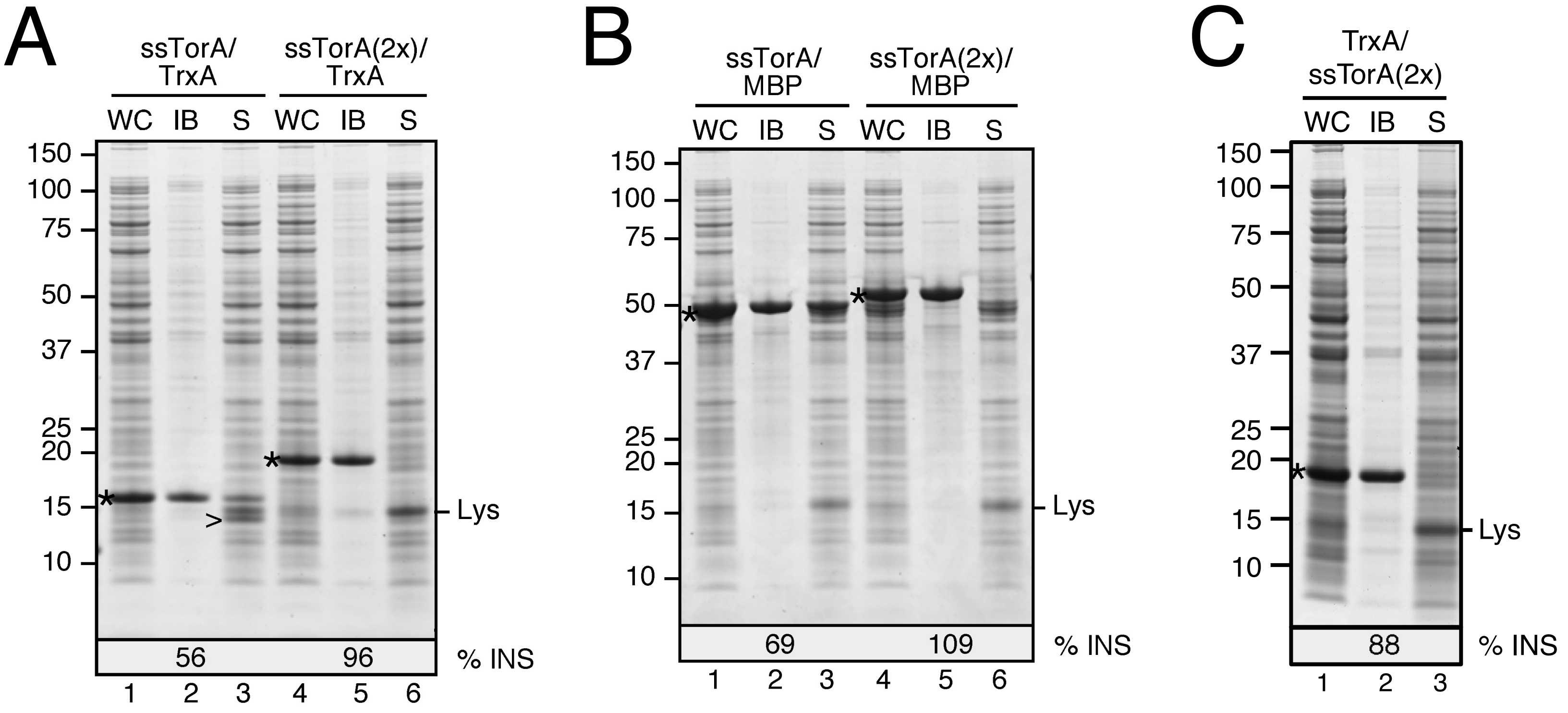

Fig. S5. IB formation upon fusion to a dual ssTorA-tag. Cells expressing dual-ssTorA tagged model proteins were processed and analyzed as described in the legend to Fig. 4. *(A)* Cells expressing TrxA or TrxA carrying an N-terminal dual ssTorA-tag, *(B)* Cells expressing MBP or MBP carrying an N-terminal dual ssTorA-tag, *(C)* Cells expressing TrxA carrying a C-terminal dual ssTorA-tag. Full-length expression products (*), a processed product of ssTorA/TrxA (>) and lysozyme added during the fractionation procedure (Lys) are indicated. Molecular mass (kDa) markers are indicated at the left side of the panels. At the bottom of the panels the relative amount of overexpressed protein in the insoluble fraction compared to the whole cell lysates is displayed (% INS) as determined by densitometry.
