# Supplementary material for: Application of an E. coli signal sequence as a versatile inclusion body tag
Source: Microb Cell Fact. 2017 Mar 21;16:50. doi: 10.1186/s12934-017-0662-4 (PMC5359840; doi:10.1186/s12934-017-0662-4)
Supplement: Supplementary file 6 — Additional file 6: Figure S6. ssTorA-mediated inclusion body formation in E. coli K-12 and B strains. [file 12934_2017_662_MOESM6_ESM.pdf]

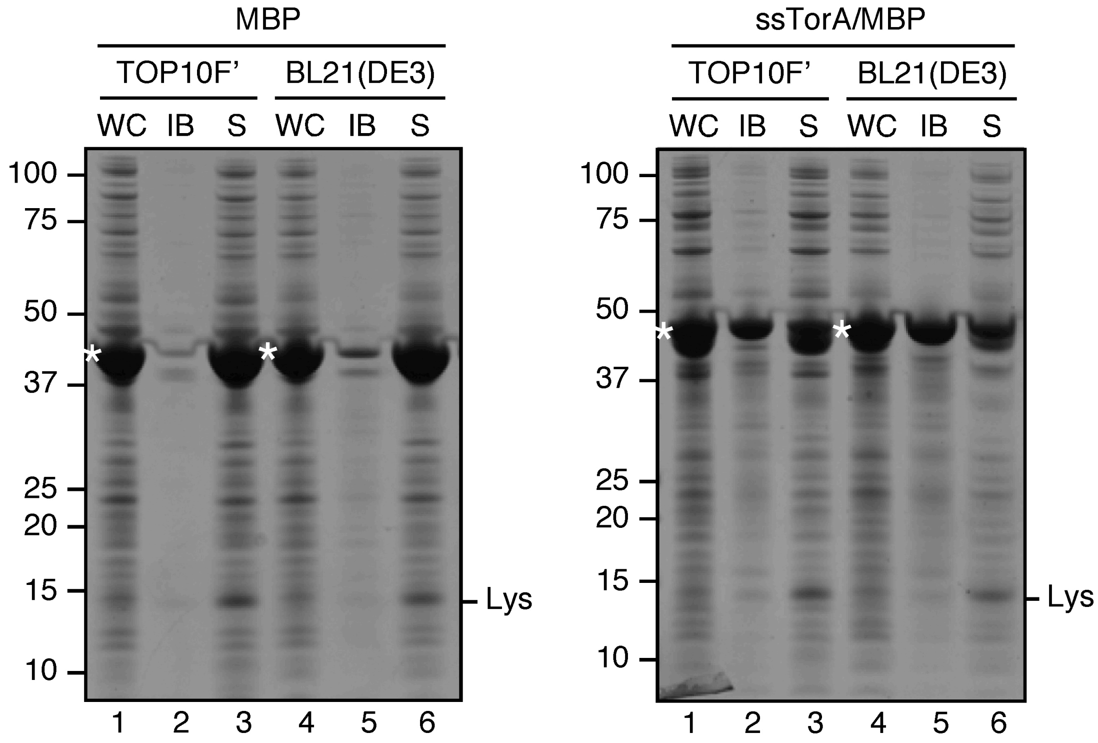

Fig. S6. ssTorA-mediated inclusion body formation in E. coli K-12 and B strains. Inclusion body formation of MBP and ssTorA/MBP in *E. coli* TOP10F’ *(A)* or BL21(DE3) *(B)* analyzed and displayed as described in the legend to Fig. 4.
