# Supplementary material for: Application of an E. coli signal sequence as a versatile inclusion body tag
Source: Microb Cell Fact. 2017 Mar 21;16:50. doi: 10.1186/s12934-017-0662-4 (PMC5359840; doi:10.1186/s12934-017-0662-4)
Supplement: Supplementary file 7 — Additional file 7: Figure S7. Expression of a ssTorA(3x)/GFP fusion protein yields fluorescent IBs. [file 12934_2017_662_MOESM7_ESM.pdf]

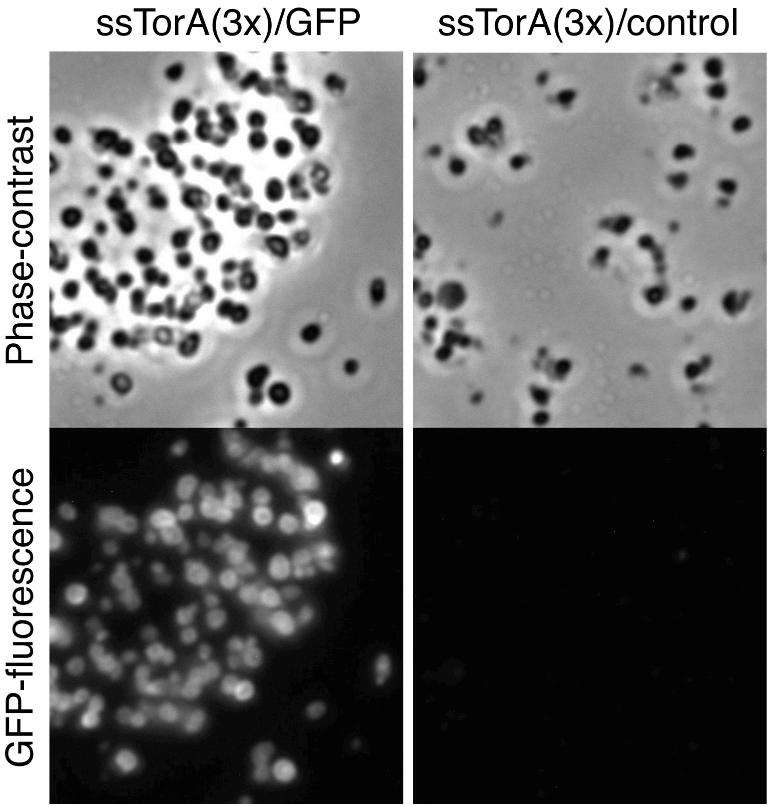

Fig. S7. Expression of a ssTorA(3x)/GFP fusion protein yields fluorescent IBs. Analysis of IBs isolated from *E. coli* TOP10F' expressing either a fusion protein comprising ssTorA(3x) and GFP (ssTorA/GFP) or a fusion protein comprising ssTorA(3x) and an unrelated control protein (ssTorA/control). After isolation, IBs were washed with Triton X-100, urea and high-salt as described in *Methods*. IBs were analyzed by phase-contrast (*top*) or fluorescence (*bottom*) microscopy using an Olympus BH2 microscope. GFP fluorescence was visualized using the BH2-RFCA IB filter cube (exciter filter BP495, dichroitic mirror DM505, barrier filter O515IF).
