# Supplementary material for: Application of an E. coli signal sequence as a versatile inclusion body tag
Source: Microb Cell Fact. 2017 Mar 21;16:50. doi: 10.1186/s12934-017-0662-4 (PMC5359840; doi:10.1186/s12934-017-0662-4)
Supplement: Supplementary file 8 — Additional file 8: Figure S8. Inclusion body formation upon expression of full-length TorA. [file 12934_2017_662_MOESM8_ESM.pdf]

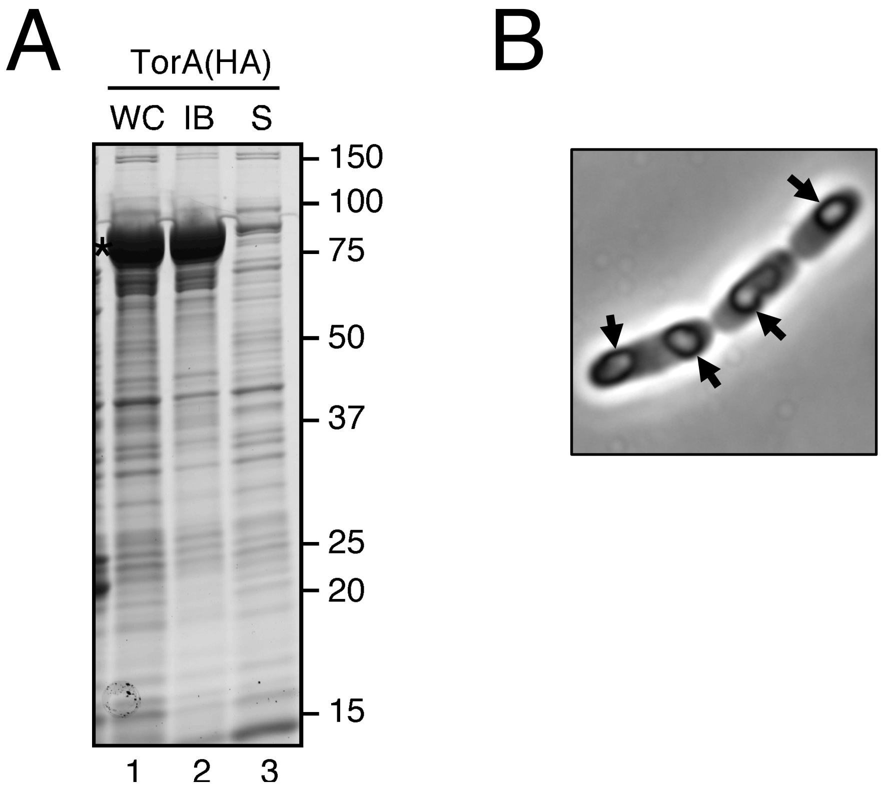

Fig. S8. Inclusion body formation upon expression of full-length TorA. A construct encoding the full-length TorA protein carrying and a small C-terminally fused HA detection tag (YPYDVPDYA), was cloned under *araBAD* promoter control in vector pBAD24 and expressed in *E. coli* TOP10F' cells. The cells were grown to an OD_{660} of 0.3 after which TorA(HA) expression was induced by addition of 0.2% of arabinose. After two hours cells were collected and inclusion body formation of TorA(HA) was analyzed *(A)* using the IB- spin down assay as described in the legend to Fig. 4 and *(B)* phase-contrast microscopy. For microscopy analysis cells were resuspended in LB medium and photographed with an Olympus F-view II camera mounted on an Olympus BH-2 microscope through an DApo100UV PL 1.30 oil 160/0.17 objective. IBs are indicated with arrows.
