# Supplementary material for: Application of an E. coli signal sequence as a versatile inclusion body tag
Source: Microb Cell Fact. 2017 Mar 21;16:50. doi: 10.1186/s12934-017-0662-4 (PMC5359840; doi:10.1186/s12934-017-0662-4)
Supplement: Supplementary file 9 — Additional file 9: Figure S9. Inclusion body formation upon expression of Hbp. [file 12934_2017_662_MOESM9_ESM.pdf]

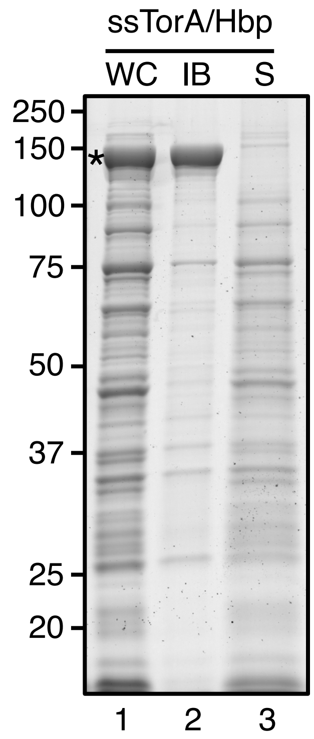

Fig. S9. Inclusion body formation upon expression of Hbp. A construct was created encoding a fusion of ssTorA to the N-terminus of a signal peptide-lacking derivative of the autotransporter protein Hbp that is normally unstable upon expression in *E. coli* [1]. The resulting ssTorA/Hbp encoding construct was cloned under lacUV5 promoter control in vector pEH3 and expressed in *E. coli* TOP10F' cells. The cells were grown to an OD_{660} of 0.3 after which ssTorA/Hbp expression was induced by addition of 1 mM of Isopropyl β-D-1-thiogalactopyranoside (IPTG). After two hours, cells were collected and inclusion body formation of ssTorA/Hbp was analyzed using the IB- spin down assay as described in the legend to Fig. 4.

Reference

1. Sijbrandi, R *et al.*: **Signal recognition particle (SRP)-mediated targeting and Sec-dependent translocation of an extracellular *Escherichia coli* protein.** *J Biol Chem* 2003, **278(7):**4654-9.
