# Supplementary material for: Application of an E. coli signal sequence as a versatile inclusion body tag
Source: Microb Cell Fact. 2017 Mar 21;16:50. doi: 10.1186/s12934-017-0662-4 (PMC5359840; doi:10.1186/s12934-017-0662-4)
Supplement: Supplementary file 10 — Additional file 10: Table S1. Plasmids used in this study. [file 12934_2017_662_MOESM10_ESM.pdf]

Table S1. Plasmids used in this study

Plasmid name	Description	Reference
pASK-IBA3	Expression vector; *tetA* promoter	IBA GmbH, Germany
pIBA-hEGF	pASK-IBA3 + *hEGF*	This work
pIBA-ssHbp/hEGF	pASK-IBA3 + *sshbp/hegf*	This work
pIBA-ssTorA/hEGF	pASK-IBA3 + *sstorA/hegf*	This work
pIBA-ssPhoE/hEGF	pASK-IBA3 + *ssphoE/hegf*	This work
pIBA-ssDsbA/hEGF	pASK-IBA3 + *ssdsbA/hegf*	This work
pIBA-ssHbp/Pla2	pASK-IBA3 + *sshbp/pla2*	This work
pIBA-ssTorA/Pla2	pASK-IBA3 + *sstorA/pla2*	This work
pIBA-ssPhoE/Pla2	pASK-IBA3 + *ssphoE/pla2*	This work
pIBA-ssDsbA/Pla2	pASK-IBA3 + *ssdsbA/pla2*	This work
pIBA-ssHbp/IL3	pASK-IBA3 + *sshbp/il3*	This work
pIBA-ssTorA/IL3	pASK-IBA3 + *sstorA/il3*	This work
pIBA-ssPhoE/IL3	pASK-IBA3 + *ssphoE/il3*	This work
pIBA-ssDsbA/IL3	pASK-IBA3 + *ssdsbA/il3*	This work
pIBA-TrxA	pASK-IBA3 + *trxA*	This work
pIBA-ssTorA/TrxA	pASK-IBA3 + *sstorA/trxA*	This work
pIBA-ssTorA/TrxA[2x]	pASK-IBA3 + *sstorA[2x]/trxA*	This work
pIBA-ssTorA/TrxA[3x]	pASK-IBA3 + *sstorA[3x]/trxA*	This work
pIBA-ssTrxA/ssTorA	pASK-IBA3 + *trxA/sstorA*	This work
pIBA-ssTrxA/ssTorA[2x]	pASK-IBA3 + *trxA/sstorA[2x]*	This work
pIBA-ssTrxA/ssTorA[3x]	pASK-IBA3 + *trxA/sstorA[3x]*	This work
pIBA-MBP	pASK-IBA3 + *malE*	This work
pIBA-ssTorA/MBP	pASK-IBA3 + *sstorA/malE*	This work
pIBA-ssTorA[2x]/MBP	pASK-IBA3 + *sstorA[2x]/malE*	This work
pIBA-ssTorA[3x]/MBP	pASK-IBA3 + *sstorA[3x]/malE*	This work
pIBA-SymE	pASK-IBA3 + *symE*	This work
pIBA-ssTorA/SymE	pASK-IBA3 + *sstorA/symE*	This work
pBAD24	Expression vector; *araBAD* promoter	[1]
pBAD24-TorA(HA)	pBAD24 + *torA-HA*	This work
pEH3	Expression vector; *lacUV5* promoter	[2]
pEH3-ssTorA/Hbp	pEH3 + *sstora/hbp*	This work

Reference

1. Guzman LM, Belin D, Carson MJ, Beckwith J: **Tight regulation, modulation, and high-level expression by vectors containing the arabinose PBAD promoter.** *J Bacteriol* 1995, **177:**4121-4130.
2. Hashemzadeh-Bonehi L, Mehraein-Ghomi F, Mitsopoulos C, Jacob JP, Hennessey ES, Broome-Smith JK: **Importance of using lac rather than ara promoter vectors for modulating the levels of toxic gene products in Escherichia coli.** *Mol Microbiol* 1998, **30:**676-678.
