# Supplementary material for: Application of an E. coli signal sequence as a versatile inclusion body tag
Source: Microb Cell Fact. 2017 Mar 21;16:50. doi: 10.1186/s12934-017-0662-4 (PMC5359840; doi:10.1186/s12934-017-0662-4)
Supplement: Supplementary file 11 — Additional file 11: Table S2. Primers used in this study. [file 12934_2017_662_MOESM11_ESM.pdf]

Table S2. Primers used in this study

Primer name	Primer sequence (5’ → 3’)	Features
ASAA_hEGF_fw	cagga**gctagc**gcagcgaattctgattctgagtgtcca	*Nhe*I site in bold; Ala-Ser-Ala-Ala coding sequence underlined
HindIII_hEGF_rev	ctag**aagctt**atctcaattcccacc	*Hin*dIII site in bold
IBA_XbaI_Hbp_fw	ctag**tctaga**taacgagggcaaaaaatgaacagaatttattctcttcg	*Xba*I site in bold; pASK-IBA3 ribosome binding site
ASAA_Hbp_rev	cgctgc**gctagc**tcctgcagagaatagtaccg	*Nhe*I site in bold; complementary Ala-Ser-Ala-Ala coding sequence underlined
IBA_XbaI_TorA_fw	ctag**tctaga**taacgagggcaaaaaatgaacaataacgatctctttc	*Xba*I site in bold; pASK-IBA3 ribosome binding site
ASAA_TorA_rev	cgctgc**gctagc**acgtcgcggcgttaacaatg	*Nhe*I site in bold; complementary Ala-Ser-Ala-Ala coding sequence underlined
IBA_XbaI_PhoE_fw	ctag**tctaga**taacgagggcaaaaaatgaaaaagagcactctggc	*Xba*I site in bold; pASK-IBA3 ribosome binding site
ASAA_PhoE_rev	cgctgc**gctagc**agatgcagatgccacaatgc	*Nhe*I site in bold; complementary Ala-Ser-Ala-Ala coding sequence underlined
IBA_XbaI_DsbA_fw	ctag**tctaga**taacgagggcaaaaaatgaaaaagatttggctggcg	*Xba*I site in bold; pASK-IBA3 ribosome binding site
ASAA_DsbA_rev	cgctgc**gctagc**gctaaacgctaaaactaaaccag	*Nhe*I site in bold; complementary Ala-Ser-Ala-Ala coding sequence underlined
ASA_PLA2_fw	cagga**gctagc**gcaggcttgctggacctaaaatc	*Nhe*I site in bold; Ala-Ser-Ala coding sequence underlined
PLA2_HindIII_rev	ctag**aagctt**tcaggagcagaggatgttg	*Hin*dIII site in bold
ASA_IL3 fw	cagga**gctagc**gcagctcccatgacccagacaa	*Nhe*I site in bold; Ala-Ser-Ala coding sequence underlined
IL3_HindIII_rev	ctag**aagctt**cagctcaaagtcgtctgttg	*Hin*dIII site in bold
XbaI_RBS_TrxA fw	gtca**tctaga**taacgagggcaaaaaatgtctgataaaattattcacctgactgac	*Xba*I site in bold; pASK-IBA3 ribosome binding site
HindIII_TrxA rev	agcc**aagctt**acgccaggttag	*Hin*dIII site in bold
ASA_TrxA_fw	gacgt**gctagc**gcatctgataaaattattcacctgactgac	*Nhe*I site in bold; Ala-Ser-Ala coding sequence underlined
XbaI_RBS_MBP_fw	gtca**tctaga**taacgagggcaaaaaatgaaaatcgaagaaggtaaactgg	*Xba*I site in bold; pASK-IBA3 ribosome binding site underlined
HindIII_MBP_rev	agcc**aagctt**acttggtgatacgagtctg	*Hin*dIII site in bold
ASA_MBP_fw	gacgt**gctagc**gcaaaaatcgaagaaggtaaactgg	*Nhe*I site in bold; Ala-Ser-Ala coding sequence underlined
XbaI_RBS_SymE fw	gtca**tctaga**taacgagggcaaaaaatgactgacacgcattctattgc	*Xba*I site in bold
HindIII_SymE rev	agcc**aagctt**acgcgactttctgtttacc	*Hin*dIII site in bold
ASA_SymE_fw	gacgt**gctagc**gcaactgacacgcattctattgc	*Nhe*I site in bold; Ala-Ser-Ala coding sequence underlined
C_ssTorA_TrxA_rv	gaaagagatcgttattgttcatcgccaggttagcgtcgag	complementary ssTorA-encoding sequence underlined
C_ssTorA_fw	atgaacaataacgatctctttc	
HindIII_STOP_ssTorA_rv	gtcac**aagctt**atgcgctagcacgtcgcgg	*Hin*dIII site in bold; Ala-Ser-Ala coding sequence underlined; STOP codon in italics
NheI_ssTorA_rep_fw	tgcat**gctagc**atgaacaataacgatctctttc	*Nhe*I site in bold
NheI_ssTorA_rep_rev	cgtga**gctagc**acgtcgcggcg	*Nhe*I site in bold
SpeI_ssTorA_rep_fw	tgcat**actagt**atgaacaataacgatctctttc	*Spe*I site in bold
XbaI_ssTorA_hisSUMO fw	gacgt**tctaga**gcaatgggcagcagccatcatc	*Xba*I site in bold
HindIII-STOP-SUMO rv	agcc**aagctt**aaccaccaatctgttctctgtg	*Hin*dIII site in bold
NheI-GFP fw	tgac**gctagc**agtaaaggagaagaacttttcac	*Nhe*I site in bold
HindIII-GFP rv	agcc**aagctt**atttgtatagttcatccatgccatg	*Hin*dIII site in bold
TorA_EcoRI_fw	gccg**gaattc**taatatgaacaataacgatctctttc	*Eco*RI site in bold
TorA-HA_SalI_rev	actg**gtcgac**tcacgcatagtcaggaacatcgtatgggta*gccgcctgg*tgatttcacctgcgacgcg	*Sal*I site in bold; HA-tag coding sequence underlined; Pro-Gly-Gly spacer coding sequence in italics
pEH_XbaI-TorA fw	taactt**tctaga**ttacaaaacttaggagggtttttaccatgaacaataacgatctctttcag	*Xba*I in bold; pEH3 ribosome binding site underlined
ssTorA(Hbp) rv	gagttcattattgaccgttcccgcagtcgcacgtcgcg	complementary Hbp-encoding sequence underlined
Hbp(ssTorA) fw	cgcgacgtgcgactgcgggaacggtcaataatgaactc	complementary ssTorA-encoding sequence underlined
Hbp(mat) rv	gctaatcatttcgcaattctgatagg	
