# Supplementary material for: Application of an E. coli signal sequence as a versatile inclusion body tag
Source: Microb Cell Fact. 2017 Mar 21;16:50. doi: 10.1186/s12934-017-0662-4 (PMC5359840; doi:10.1186/s12934-017-0662-4)
Supplement: Supplementary file 12 — Additional file 12. Supplement methods. [file 12934_2017_662_MOESM12_ESM.pdf]

# Supplemental Methods

*Plasmid construction*

The plasmids used in this study are outlined in Table S1 (*Additional file 10*). The primers used for cloning are displayed in Table S2 (*Additional file 11*).

Unless stated otherwise, proteins were expressed under control of a *tetA* promoter from vector pASK-IBA3 (IBA GmbH). In order to express fusion proteins comprising a signal sequence and hEGF, a chimera encoding ssHbp/hEGF was initially created *via* a three-round overlap-extension PCR procedure. In the first round, the gene encoding recombinant hEGF was amplified using pUC19-hEGF(3SS), which carries a synthetic hEGF-encoding sequence (Genbank accession number AF274587 with the following silent mutations: A15G; T30C and T75A) (gift from W. Quax, University of Groningen, The Netherlands), as a template. The forward primer used was ASAA_hEGF_fw and the reverse primer was *Hin*dIII_hEGF_rev. In the second round of PCR, the ssHbp coding sequence was amplified using pEH3-Hbp [1] as a template. The forward primer used was IBA_*Xba*I_Hbp_fw and the reverse primer used was ASAA_Hbp_rev. The ssHbp and hEGF coding sequences were fused in a third round of PCR. In this reaction the products from PCR rounds 1 and 2 were mixed and combined with the primers IBA_*Xba*I_Hbp_fw and reverse primer *Hin*dIII_hEGF_rev. The resulting product was cloned into pASK-IBA3 using the *Xba*I and *Hin*dIII restriction sites, generating pIBA-ssHbp/hEGF.

To create the remaining signal sequence/hEGF fusion constructs, cassettes encoding the signal sequences of TorA, PhoE, and DsbA were amplified by PCR. To generate the ssTorA-encoding cassette pC4Meth-100TorA/P2 [2] was used as a template in combination with primers IBA_*Xba*I_TorA_fw and ASAA_TorA_rev. The ssPhoE-encoding cassette was generated using pC4Meth-94PhoE [3] as the template and the primers IBA_*Xba*I_PhoE_fw and ASAA_PhoE_rev. To generate the ssDsbA cassette, *E. coli* TOP10F' genomic DNA was used as the template with the primers IBA_*Xba*I_DsbA_fw and ASAA_DsbA_rev. The resulting products were cloned into pIBA-Hbp/hEGF using the *Xba*I and *Nhe*I restriction sites, giving rise to pIBA-ssTorA/hEGF, pIBA-ssPhoE/hEGF and pIBA-ssDsbA/hEGF, respectively.

In order to create pIBA-signal sequence/PLA2 constructs, a fragment encoding the mature region of human PLA2(TypeV) was amplified by PCR. The template used was pET24as/PLA2V (gift from P. Cronet, Astrazeneca) carrying a human PLA2(TypeV) encoding sequence (Genbank accession number NM_00029). The primers used were ASA_PLA2_fw and PLA2_*Hin*dIII_rev. The resulting product was cloned into pIBA-ssHbp/hEGF, pIBA-ssTorA/hEGF, pIBA-ssPhoE/hEGF and pIBA-ssDsbA/hEGF using the *Nhe*I and *Hind*III restriction sites, giving rise to pIBA-ssHbp/PLA2, pIBA-ssTorA/PLA2, pIBA-ssPhoE/PLA2 and pIBA-ssDsbA/PLA2, respectively.

In order to create pIBA-signal sequence/IL3 constructs, a fragment encoding the mature region of human IL3 was amplified by PCR using pLat-IL3 [4] as a template. The primers used were ASA_IL3 fw and IL3_HindIII_rev. The resulting product was cloned into pIBA-ssHbp/hEGF, pIBA-ssTorA/hEGF, pIBA-ssPhoE/hEGF and pIBA-ssDsbA/hEGF using the *Nhe*I and *Hin*dIII restriction sites, giving rise to pIBA-ssHbp/IL3, pIBA-ssTorA/IL3, pIBA-ssPhoE/IL3 and pIBA-ssDsbA/IL3, respectively.

Plasmids pIBA-TrxA, pIBA-ssTorA/TrxA, pIBA-MBP, pIBA-ssTorA/MBP, pIBA-SymE and pIBA-ssTorA/SymE were all constructed by PCR. To construct pIBA-TrxA plasmid pMO2 [5] as a template along with the primers XbaI-RBS-TrxA fw and HindIII-TrxA rev. The resulting PCR fragment was cloned into plasmid pIBA-ssTorA/Pla2 using the *Xba*I and *Hin*dIII restriction sites, generating pIBA-TrxA. For construction of pIBA-ssTorA/TrxA plasmid pMO2 was used as a template. The primers used were ASA_TrxA_fw and *Hin*dIII_TrxA_rev. The resulting PCR fragment was cloned into plasmid pIBA-ssTorA/Pla2 using the *Nhe*I and *Hin*dIII restriction sites, giving rise to pIBA-ssTorA/TrxA. To construct pIBA-MBP *E. coli* MG1655 genomic DNA was used as a template along with the primers XbaI_RBS_MBP_fw carrying an *Xba*I site and the and *Hin*dIII_MBP_rev. The PCR fragment was cloned into plasmid pIBA-ssTorA/Pla2 using the *Xba*I and *Hin*dIII restriction sites, giving rise to pIBA-MBP. To create the plasmid pASK-IBA-TorA/MBP, *E. coli* MG1655 genomic DNA was used as a template and the primers used were ASA_MBP fw and HindIII_MBP rev. The resulting PCR fragment was cloned into plasmid pIBA-ssTorA/Pla2 using the NheI and HindIII restriction sites, resulting in pIBA-ssTortA/MBP. To create pIBA-SymE, *E. coli* MG1655 genomic DNA was used as the template along with the primers *Xba*I-RBS-SymE fw and *Hin*dIII_SymE rev. The resulting fragment was cloned into pIBA-

ssTorA/Pla2 using *Xba*I/*Hin*dIII sites, generating pIBA-SymE. To create pIBA-ssTorA/SymE, *E. coli* MG1655 genomic DNA was used as the template. The primers used were ASA_SymE_fw and HindIII_SymE_rev. The resulting PCR product was cloned into pIBA-ssTorA/PLA2 using the *Nhe*I/*Hin*dIII sites, giving rise to pIBA-ssTorA/SymE.

Plasmid pIBA-TrxA/ssTorA was created using a three-step overlap-extension PCR procedure. In the first step, the gene encoding TrxA was amplified using pIBA-ssTorA/TrxA as a template plus the primers *Xba*I_RBS_TrxA_fw and C_ssTorA_TrxA_rv. In the second step, the ssTorA coding sequence was amplified using pIBA-ssTorA/TrxA as a template along with the primers C_ssTorA_fw and *Hin*dIII_STOP_ssTorA_rv. In the third step, the TrxA and ssTorA encoding sequences were fused. In this reaction the products from PCRs 1 and 2 were mixed and combined with the primers *Xba*I_RBS_TrxA_fw and *Hin*dIII_STOP_ssTorA_rv. The resulting product was cloned into pIBA-TrxA using the *Xba*I/*Hin*dIII sites, resulting in pIBA-TrxA/ssTorA.

Plasmids encoding fusions carrying two or three ssTorA sequences at the N-terminus of the target protein were created by PCR as follows. The ssTorA encoding sequence was amplified with flanking *Nhe*I sites using pASK-IBA-ssTorA/Pla2 as a template. The primers used were ASA_*NheI*_ssTorA_rep_fw and ASA_*NheI*_ssTorA_rep_rev. The resulting fragment (*Nhe*I-ssTorA-*Nhe*I) was cloned into the single *Nhe*I site of pIBA-TorA/TrxA, generating pIBA-ssTorA(2x)/TrxA, carrying two ssTorA coding sequences in frame with the TrxA coding sequence. Cloning of the fragment *Nhe*I-ssTorA-NheI into the single *Nhe*I site of pIBA-ssTorA/MBP yielded pIBA-ssTorA(2x)/MBP and pIBA-ssTorA(3x)/MBP, carrying two or three ssTorA coding sequences in frame with the MBP coding sequence, respectively. To create a pIBA-ssTorA/TrxA derivative carrying three ssTorA-encoding sequences, an ssTorA-encoding PCR fragment was generated carrying flanking *Spe*I and *Nhe*I sites (*Spe*I-ssTorA-*Nhe*I). Plasmid pIBA-ssTorA/TrxA was used as a template along with the primers ASA_*Spe*I_ssTorA_rep_fw and ASA_*Nhe*I_ssTorA_rep_rev. The resulting product was digested with *Spe*I and *Nhe*I and cloned into the single *Nhe*I site of pIBA-ssTorA/TrxA, yielding pIBA-ssTorA(3x;SN)/TrxA. To create plasmids carrying two or three ssTorA sequences at the C-terminus of TrxA, the digested *Spe*I-ssTorA-*Nhe*I fragment was cloned into the *Nhe*I site of pIBA-TrxA/ssTorA, yielding pIBA-TrxA/ssTorA(2x) and pIBA-TrxA/ssTorA(3x), carrying two or three ssTorA coding sequences in frame with the TrxA coding sequence, respectively.

In order to create plasmid pIBA-ssTorA(3x)/GFP, first plasmid pIBA-ssTorA(3x)/SUMO was created by PCR using pET-SUMO (Invitrogen) as a template. The primers used were *Xba*I_ssTorA_hisSUMO fw and *Hin*dIII-STOP-SUMO rv. The resulting product was digested with *Xba*I and *Hin*dIII and cloned into *Nhe*I/*Hin*dIII-digested pIBA-ssTorA(3x;SN)/TrxA, yielding pIBA-ssTorA(3x)/SUMO. Subsequently, the GFPmut2-encoding region was amplified by PCR using pTHV038 [6] as a template in combination with the primers *Nhe*I-GFP fw and *Hin*dIII-GFP rv. The resulting product was cloned into pIBA-ssTorA(3x)/SUMO using the *Nhe*I/*Hin*dIII restriction sites, yielding pIBA-ssTorA(3x)/GFP.

Plasmid pBAD24-TorA(HA) was constructed by PCR using *E. coli* MG1655 genomic DNA as the template and the primers TorA_*Eco*RI_fw and TorA-HA_*Sal*I-rev. Part of the PCR product (*torA**) was digested with *Eco*RI and the resulting 2306 bp fragment was cloned into the single EcoRI site of pBAD24 [7], yielding pBAD24-TorA(HA)-E/E. Subsequently, part of the PCR product *torA** was digested with *Sfu*I and *Sal*I generating a 2354 bp fragment that was cloned into the *Sfu*I/*Sal*I sites of pBAD24-TorA(HA)-E/E, yielding pBAD24-TorA(HA).

Plasmid pEH3-ssTorA/Hbp was created using a three-round PCR procedure. First a PCR was carried out using pBAD24-TorA(HA) as a template and the primers pEH_XbaI-TorA fw and ssTorA(Hbp) rv. Second, a PCR was carried out using pEH3-Hbp [1] as a template and the primers Hbp(ssTorA) fw and Hbp(mat) rv. Third, a fusion PCR was carried out using the products from step 1 and 2 as templates together with the primers pEH_XbaI-TorA fw and Hbp(mat) rv. The resulting product was cloned into the *Xba*I/*Xma*I sites of pEH3-Hbp, yielding pEH3-ssTorA/Hbp. All DNA sequences were confirmed using semi-automated DNA sequencing.

## References


1. Jong WS, ten Hagen-Jongman CM, den Blaauwen T, Slotboom DJ, Tame JR, Wickstrom D, de Gier JW, Otto BR, Luirink J: **Limited tolerance towards folded elements during secretion of the autotransporter Hbp.** *Mol Microbiol* 2007, **63:**1524-1536.
2. Jong WS, ten Hagen-Jongman CM, Genevaux P, Brunner J, Oudega B, Luirink J: **Trigger factor interacts with the signal peptide of nascent Tat substrates but does not play a critical role in Tat-mediated export.** *Eur J Biochem* 2004, **271:**4779-4787.
3. Valent QA, de Gier JW, von Heijne G, Kendall DA, ten Hagen-Jongman CM, Oudega B, Luirink J: **Nascent membrane and presecretory proteins synthesized in Escherichia coli associate with signal recognition particle and trigger factor.** *Mol Microbiol* 1997, **25:**53-64.
4. Westers L, Dijkstra DS, Westers H, van Dijl JM, Quax WJ: **Secretion of functional human interleukin-3 from Bacillus subtilis.** *J Biotechnol* 2006, **123:**211-224.

5. Huber D, Boyd D, Xia Y, Olma MH, Gerstein M, Beckwith J: **Use of thioredoxin as a reporter to identify a subset of Escherichia coli signal sequences that promote signal recognition particle-dependent translocation.** *J Bacteriol* 2005, **187:**2983-2991.
6. Den Blaauwen T, Aarsman ME, Vischer NO, Nanninga N: **Penicillin-binding protein PBP2 of Escherichia coli localizes preferentially in the lateral wall and at mid-cell in comparison with the old cell pole.** *Mol Microbiol* 2003, **47:**539-547.
7. Guzman LM, Belin D, Carson MJ, Beckwith J: **Tight regulation, modulation, and high-level expression by vectors containing the arabinose PBAD promoter.** *J Bacteriol* 1995, **177:**4121-4130.
